# Expression and Function of Different Guanine-Plus-Cytosine Content 16S rRNA Genes in *Haloarcula hispanica* at Different Temperatures

**DOI:** 10.3389/fmicb.2017.00482

**Published:** 2017-03-28

**Authors:** Yu Sato, Taketomo Fujiwara, Hiroyuki Kimura

**Affiliations:** ^1^Department of Environment and Energy Systems, Graduate School of Science and Technology, Shizuoka UniversityShizuoka, Japan; ^2^Department of Biological Science, Faculty of Science, Shizuoka UniversityShizuoka, Japan; ^3^Department of Geosciences, Faculty of Science, Shizuoka UniversityShizuoka, Japan; ^4^Research Institute of Green Science and Technology, Shizuoka UniversityShizuoka, Japan

**Keywords:** 16S rRNA genes, guanine-plus-cytosine content, *Haloarcula*, temperature, intragenomic heterogeneity

## Abstract

The halophilic archaeon *Haloarcula hispanica* harbors three ribosomal RNA (rRNA) operons (*rrnA*, *rrnB*, and *rrnC*) that contain the 16S rRNA genes *rrsA*, *rrsB*, and *rrsC*, respectively. Although *rrsB* and *rrsC* (*rrsBC*) have almost identical sequences, the *rrsA* and *rrsBC* sequences differ by 5.4%, and they differ by 2.5% with respect to guanine-plus-cytosine content (*P*_GC_). The strong correlation between the typical growth temperatures of archaea and *P*_GC_ of their 16S rRNA genes suggests that *H. hispanica* may harbor different 16S rRNA genes having different *P*_GC_ to maintain rapid growth in a wide range of temperatures. We therefore performed reverse transcription-coupled quantitative PCR to assess expression levels of *rrsA* (*P*_GC_, 58.9%) and *rrsBC* (*P*_GC_, 56.4–56.5%) at various temperatures. The expression ratio of *rrsA* to *rrsBC* increased with culture temperature. Mutants with complete deletions of one or two of the three rRNA operons were constructed and their growth rates at different temperatures compared to that of the wild-type. The growth characteristics of the rRNA operon single-mutant strains were indistinguishable from the wild-type. The rRNA operon double-mutant strains maintained the same temperature range as wild-type but displayed reduced growth rates. In particular, the double-mutant strains grew much slower than wild-type at low temperature related to minimum growth temperature of the wild-type. On the other hand, at physiologically high temperatures the wild-type and the double-mutant strain which harbors only *rrnA* with high-*P*_GC_ *rrsA* grew significantly faster than the double-mutant strain which harbors only *rrnC* with low-*P*_GC_ *rrsC*. These findings suggest the importance of 16S rRNAs transcribed from *rrsA* with high-*P*_GC_ in maintaining rapid growth of this halophilic archaeon at raised growth temperatures.

## Introduction

Ribosomal RNA (rRNA) genes, especially 16S rRNA genes, are a particularly good marker for phylogenetic analysis of prokaryotes because they are highly conserved in all prokaryotes (Staley, 2002). Many studies have examined the phylogenetic positions of prokaryotic species and microbial diversities in natural environments using 16S rRNA gene sequences (e.g., Hiraoka et al., 2016; Kuechler et al., 2016). On the other hand, recent studies based on genome sequences have demonstrated that the genomes of certain prokaryotes harbor several divergent 16S rRNA genes. Sun et al. (2013) reported that almost half of the complete prokaryotic genomes examined contain a base or more dissimilarity in 16S rRNA genes. Furthermore, >3% differences in 16S rRNA gene sequences have been detected in 14 of the 1690 complete genomes in domain Bacteria (Větrovský and Baldrian, 2013). Within the domain Archaea, some methanogens, e.g., *Methanocaldococcus jannaschii* and *Methanothermobacter thermoautotrophicus*, exhibit 16S rRNA gene polymorphisms displaying as much as 0.1% divergence, whereas some halophilic archaea harbor more divergent 16S rRNA gene copies (4.9–9.8%) in their genomes (Acinas et al., 2004; Boucher et al., 2004; Pei et al., 2010; Sun et al., 2013).

*Haloarcula* strains, halophilic archaea belonging to class *Halobacteria*, have mainly been isolated from solar salterns and salt lakes (Torreblanca et al., 1986), where large day-night and seasonal temperature variations are observed (Post, 1977; Wieland et al., 2005; Sima et al., 2013; Andrade et al., 2015). So far, 15 *Haloarcula* strains, including 10 strains named validly, have been reported (Javor et al., 1982; Mizuki et al., 2004; Namwong et al., 2011; Ding et al., 2014; Yun et al., 2015). Complete genome sequences of four strains, i.e., *H. hispanica*, two strains closely related to *H. hispanica*, and *H. marismortui*, have been determined and show that all strains carry three rRNA operons (*rrnA*, *rrnB*, and *rrnC*) (Baliga et al., 2004; Liu et al., 2011; Ding et al., 2014; Yun et al., 2015). Each rRNA operon includes the 5S rRNA gene, the 16S rRNA gene (*rrsA, rrsB*, or *rrsC*), and the 23S rRNA gene. The 5S rRNA genes have identical sequences. Although the 23S rRNA gene sequences of *rrnA* and *rrnB* are almost identical, the 23S rRNA gene sequence of *rrnC* is slightly different (∼2%) from those of *rrnA* and *rrnB*. Among the 16S rRNA genes, the *rrsB* and *rrsC* (*rrsBC*) sequences are almost identical, whereas the *rrsA* and *rrsBC* sequences differ by ∼6% (Dennis et al., 1998; Dennis, 1999).

Previous studies of the expression of 16S rRNA genes in *Haloarcula* strains under different salinity conditions suggest that variation in salinity does not affect the expression level of each 16S rRNA gene (López-López et al., 2007; Cui et al., 2009). On the other hand, López-López et al. (2007) demonstrated that *H. marismortui* displays different expression patterns of each 16S rRNA gene under a wide range of temperatures. They also performed cultivation experiments with wild-type *H. marismortui* and the rRNA operon single-mutant strain, which lacks *rrnB* containing low guanine-plus-cytosine content (*P*_GC_) of 16S rRNA gene, under various temperature conditions (Tu et al., 2005; López-López et al., 2007). They found that growth of the rRNA operon single-mutant strain was slower than that of wild-type at all tested temperatures. López-López et al. (2007) could not determine whether *rrnB* inactivation or a lower copy number of rRNA operons would affect growth of the mutant strain, because the rRNA operon double-mutant strains that harbor only one rRNA operon containing low- or high-*P*_GC_ 16S rRNA gene were not constructed and examined. Therefore, the functional importance of rRNA transcribed from each rRNA operon including *rrsA*, *rrsB*, or *rrsC* on growth under different temperature conditions has not been well understood yet.

Previous studies have reported that 16S rRNA gene sequences are naturally inscribed with the thermal features of their prokaryotic hosts (Galtier and Lobry, 1997; Khachane et al., 2005; Kimura et al., 2007, 2010, 2013). The observation was based on a high correlation between the growth temperatures of the prokaryotes and the *P*_GC_ of their 16S rRNA sequences: 16S rRNA genes of hyperthermophiles and thermophiles tend to have high *P*_GC_, whereas 16S rRNA genes of mesophiles and psychrophiles have relatively low *P*_GC_. On the basis of the relationship between the growth temperatures and *P*_GC_ of 16S rRNA gene sequences, we propose that *Haloarcula* strains express and utilize high *P*_GC_ of 16S rRNAs at high temperature and low *P*_GC_ of 16S rRNAs at low temperature, respectively.

In the present study, *Haloarcula hispanica*, for which the complete genome sequence has been determined, was used. The 16S rRNA genes of *H. hispanica* were sequenced, and the minimum (*T*_min_), optimum (*T*_opt_), and maximum (*T*_max_) growth temperatures were estimated based on *P*_GC_ of the 16S rRNA genes using the microbial molecular thermometer proposed by Kimura et al. (2013). Additionally, expression levels of *rrsA* and *rrsBC* between 25°C (actual *T*_min_ of the strain) and 50°C (actual *T*_max_ of the strain) were determined by reverse transcription-coupled quantitative PCR (qPCR) using specific primer sets. Moreover, we constructed rRNA operon single-mutant strains that lack *rrnA*, *rrnB*, or *rrnC* by using wild-type *H. hispanica*. We further constructed rRNA operon double-mutant strains that harbor only *rrnA* or *rrnC* by using the single-mutant strains and assessed their growth in a wide temperature range. The combined results from both gene expression and mutation experiments provide insight into the physiological advantage of harboring 16S rRNA genes of different sequence with respect to the growth of *H. hispanica*.

## Materials and Methods

### Strain and Cultivation for DNA Extraction

*Haloarcula hispanica* JCM8911 was obtained from the Japan Collection of Microorganisms (JCM, Tsukuba, Ibaraki, Japan). The strain was grown in Medium 307, which contained 2 g casamino acid (BD, Franklin Lakes, NJ, USA), 2 g Bacto yeast extract (BD), 1 g sodium glutamate, 3 g trisodium citrate, 10 g MgSO_4_⋅7H_2_O, 1 g CaCl_2_⋅2H_2_O, 1 g KCl, 200 g NaCl, 0.36 mg FeCl_2_⋅4H_2_O, and 0.36 mg MnCl_2_⋅4H_2_O per liter of distilled water. After the pH of the medium was adjusted to 7.0, the medium was sterilized by filtration with a polyethersulfone membrane filter (pore size, 0.22 μm; Thermo Fisher Scientific, Waltham, MA, USA) and autoclaving at 121°C for 20 min. Exactly 20 ml of the medium was injected into autoclaved 60-ml screw-cap test tubes and inoculated with cells of *H. hispanica* in exponential growth phase in pre-culture, and the cultures were incubated in the darkness with shaking at 180 rpm at 37°C. The cultures were centrifuged at 6230 × *g* for 3 min. The pelleted cells were stored at -25°C until DNA extraction.

### Cloning and Sequencing of 16S rRNA Genes

In order to make standards for qPCR described below, we performed cloning and sequencing of 16S rRNA genes of *H. hispanica*. Bulk DNA was extracted from *H. hispanica* cells grown in Medium 307 with modifications (Tchinda et al., 2016). Briefly, the pelleted cells were lysed with lysozyme and proteinase K solution. Then the genomic DNA was extracted with successive phenol:chloroform:isoamyl alcohol and chloroform:isoamyl alcohol steps and precipitated with ethanol. Next, 16S rRNA genes were amplified from the bulk DNA using the archaea-specific primer set 8aF/1512uR (**Table 1**). PCR products were purified with a MicroSpin S-400 HR column (GE Healthcare, Little Chalfont, UK) and cloned using the Zero Blunt TOPO PCR Cloning kit (Life Technologies, Carlsbad, CA, USA). The PCR products were ligated into vector pCR4Blunt-TOPO (Life Technologies). *Escherichia coli* TOP10 cells (Life Technologies) were transformed with the ligated plasmid to construct a clone library. Insert DNA from selected recombinant colonies was sequenced by the dideoxy cycle-sequencing method using a Model 3730*xl* DNA analyzer (Applied Biosystems, Foster City, CA, USA). The most similar 16S rRNA gene sequence was determined by the BLAST program (Altschul et al., 1990). The 16S rRNA gene sequences obtained in this study were deposited in the DDBJ/EMBL/GenBank database under accession numbers LC085245, LC085246, and LC085247.

**Table 1:** Primers targeting 16S rRNA genes for PCR, reverse transcription-coupled qPCR, and sequencing in this study.

Name	Sequence	Position	Target	Reference
8aF	5′-TCY GGT TGA TCC TGC C-3′	3–18	Archaeal 16S rRNA gene	Burggraf et al., 1991
1512uR	5′-GGT TAC CTT GTT ACG ACT T-3′	1424–1442	Prokaryotic 16S rRNA gene	Matsushita et al., 2016
109aF	5′-AMD GCT CAG TAA CAC GT-3′	83–99	Archaeal 16S rRNA gene	Matsushita et al., 2016
915aR	5′-GTG CTC CCC CGC CAA TTC CT-3′	858–877	Archaeal 16S rRNA gene	Matsushita et al., 2016
rrsAf	5′-CGT CCA GCG GAA ACT GTC CGG-3′	569–589	partial sequence of *rrsA*	This study
rrsAr	5′-CCG TCG GGT CCG TCT TCC TGA G-3′	674–695	Partial sequence of *rrsA*	This study
rrsBCf	5′-GGC GTC CGG TGG AAA CTA CAC AG-3′	567–589	Partial sequence of *rrsBC*	This study
rrsBCr	5′-CAC TGT CGG GTC CGG TCT CTC AAC-3′	674–697	Partial sequence of *rrsBC*	This study

### Estimating *T*_min_, *T*_opt_, and *T*_max_ Based on *P*_GC_ of 16S rRNA Genes

Because thermophilic and hyperthermophilic archaea have greater *P*_GC_ values for 16S rRNA genes compared with psychrophilic and mesophilic archaea (Galtier and Lobry, 1997; Khachane et al., 2005; Kimura et al., 2006, 2007, 2010), Kimura et al. (2013) proposed linear regression equations to infer *T*_min_, *T*_opt_, and *T*_max_ of cultured and not-yet cultured archaea based on *P*_GC_ of 16S rRNA genes. We therefore used these equations to estimate growth temperatures of *H. hispanica* based on *P*_GC_ value of each 16S rRNA gene.

Kimura et al. (2013) used partial 16S rRNA gene sequences (ca. 800 bp) between the archaea-specific primers 109aF and 915aR in order to estimate their growth temperatures (**Table 1**). Thus, we manually selected the internal sequences from the 16S rRNA gene sequences determined in this study. *P*_GC_ values for the internal sequences were calculated using Genetyx-Mac ver. 17.0.6 (Genetyx, Tokyo, Japan). *T*_min_, *T*_opt_, and *T*_max_ were calculated based on *P*_GC_ of the respective sequence using Kimura’s equations.

### Culture Experiment to Assess the Expression of 16S rRNA Genes

*Haloarcula hispanica* cells in exponential growth phase in pre-culture were inoculated into 60-ml screw-capped tubes containing 20 ml of Medium 307. The cultures were incubated in the dark with shaking at 180 rpm at 25, 30, 35, 40, 45, and 50°C. The optical density at 660 nm (OD_660_) of the culture was monitored using a Spectronic 200 spectrophotometer (Thermo Fisher Scientific) with sterilized medium as the negative control. When the cultures reached the early exponential growth phase (OD_660_ = 0.25–0.50), the cultures were centrifuged at 6230 × *g* for 3 min. The pelleted cells were mixed with 100 μl RNAlater (Life Technologies) and stored at –85°C until RNA extraction.

### RNA Extraction and Complementary DNA (cDNA) Synthesis

Cells were thawed on ice, and the RNAlater was removed and discarded. Total RNA was extracted from the cells using the *mir*Vana miRNA Isolation kit (Ambion, Austin, TX, USA). Contaminating genomic DNA in the extracted RNA samples was removed using the TURBO DNA-Free kit (Life Technologies). Total RNA was purified with the RNeasy MinElute Cleanup kit (Qiagen, Hilden, Germany). The quality and concentration of RNA were verified using a 2100 Bioanalyzer (Agilent Technologies, Santa Clara, CA, USA) and a NanoVue Plus spectrophotometer (GE Healthcare). Single-strand complementary DNA (cDNA) was synthesized from the purified total RNA using the SuperScript III first strand synthesis system (Life Technologies) as following manufacturer’s protocol. The cDNA was purified using QIAquick PCR Purification kit (Qiagen). The purified cDNA was stored at -25°C until qPCR analysis.

### qPCR

The specific primer sets rrsAf/rrsAr for *rrsA* and rrsBCf/rrsBCr for *rrsBC* were designed using Primer Express 2.0 software ver. 2.0 (Applied Biosystems) (**Table 1**). To test the specificity of these primer sets, qPCR was performed with PCR products of *rrsA*, *rrsB*, and *rrsC* that were amplified from the clones in the 16S rRNA gene-clone library described above. The PCR products were purified with QIAquick PCR purification kit (Qiagen). The qPCR was performed on an ABI Prism 7300 Real Time PCR System (Applied Biosystems) with PowerUP SYBR Green master mix (Life Technologies). Since the sequences of *rrsB* and *rrsC* are nearly identical, it was not possible to design primer sets for specifically amplifying *rrsB* and *rrsC*, independently.

Next, *rrsA* and *rrsBC* in the cDNA were quantified by qPCR using the ABI Prism 7300 Real Time PCR System. Each PCR mixture contained 2 μl of diluted cDNA template, 2 μl of each designed primer set (each 300 nM), 10 μl of PowerUP SYBR Green PCR master mix (Life Technologies), and 4 μl of nuclease-free water (Ambion). The PCR conditions included an initial step of 50°C for 2 min and 95°C for 2 min followed by 40 cycles of 95°C for 15 s, 58°C for 15 s, and 72°C for 1 min. The standard curves were prepared from diluted PCR products (1/10, 1/100, 1/1000, 1/10000) of *rrsA*, *rrsB*, and *rrsC* that were amplified from the clones in the 16S rRNA gene-clone library described above. The PCR reactions were performed in triplicate for technical repeats and four individuals for biological repeats.

### Construction and Cultivation of rRNA Operon Double-Mutant Strains

**FIGURE 1:**
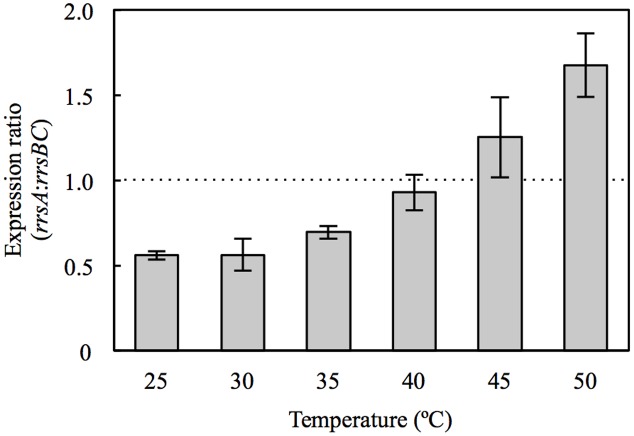
**Expression ratio of *rrsA* to *rrsBC* in *Haloarcula hispanica* cultured at 25, 30, 35, 40, 45, and 50°C**. Error bars denote standard deviation of mean values for triplicate measurements. The dashed line indicates *rrsA*:*rrsBC* ratio of 1.0.

**FIGURE 2:**
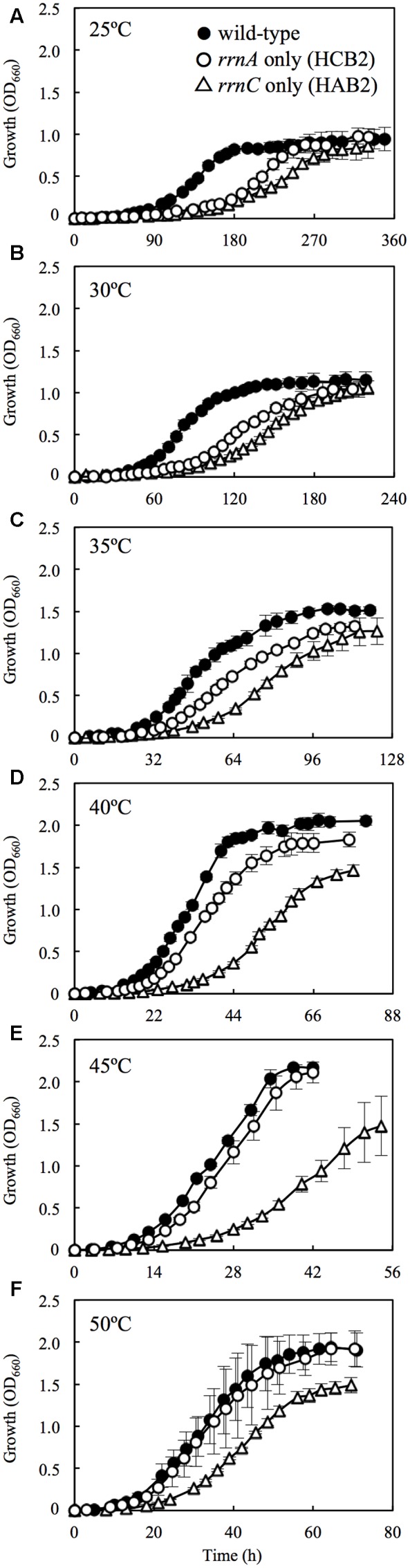
**Growth curves for wild-type *H. hispanica* and the rRNA operon double-mutant strains at 25 (A), 30 (B), 35 (C), 40 (D), 45 (E), and 50°C (F)**. Error bars denote standard deviation of mean values for quadruplicate or quintuplicate measurements.

To assess any advantage to *H. hispanica* of having multiple distinct 16S rRNA genes, we disrupted the three rRNA operons by using a mutation method (See Supplementary Materials and Methods for details). Briefly, an rRNA operon in wild-type *H. hispanica* was replaced with novobiocin resistance gene to construct rRNA operon single-mutant strains (HA2, HB2, or HC2) that lack *rrnA*, *rrnB*, or *rrnC*, respectively (Supplementary Table 1 and Figure 1). Furthermore, *rrnB* in HC2, *rrnA* in HC2 (or *rrnC* in HA2), and *rrnB* in HA2 were replaced with mevinolin resistance genes to construct the rRNA operon double-mutant strains HCB2, HCA2 (or HAC2), or HAB2 that contain only *rrnA, rrnB*, or *rrnC*, respectively (Supplementary Table 1 and Figure 2). Disruption of operons *rrnA*, *rrnB*, and *rrnC* in these mutant strains was confirmed by PCR amplification using primer sets, AVF/ACR, BVF/BCR, or CVF/CCR (Supplementary Table 2). The rRNA operon double-mutant strains harboring only *rrnB* (HCA2 and HAC2) could not be constructed despite repeated attempts (Supplementary Table 1).

The rRNA operon single- and double-mutant strains and wild-type strain were inoculated into Medium 307 and incubated in the dark with shaking at 180 rpm at 25, 30, 35, 40, 45, and 50°C. OD_660_ of the cultures was monitored using a Spectronic 200 spectrophotometer (Thermo Fisher Scientific), and growth curves were drawn based on the values. The culture experiments were performed in quadruplicate or quintuplicate. The culture experiment at 50°C (actual *T*_max_) was carried out twice to confirm the growth of the double-mutant and wild-type strains.

To calculate the maximum growth rates at each temperature, we determined a cell number factor to convert from OD_660_ value to cell density. Briefly, wild-type *H. hispanica* was grown in Medium 307, and the cultures were diluted with sterilized medium. After OD_660_ of the cultures was measured using Spectronic 200 spectrophotometer (Thermo Fisher Scientific), the cells in the cultures were fixed in formaldehyde (final concentration 7%) for 16 h at 4°C as described previously (Antón et al., 1999). The cultures were filtered using pre-blackened polycarbonate filters (pore size, 0.2 μm; diameter, 25 mm) (Millipore, Billerica, MA, USA). The cells collected on the filters were stained with SYBR Green I (1:100 dilution) (Life Technologies). The cells were observed under a model BX51 epifluorescence microscope equipped with a U-MNIB3 fluorescence filter (Olympus, Tokyo, Japan), and 50 microscopic fields were counted for each sample. A cell number factor of 2.1 × 10^9^ cells ml^-1^ per OD_660_ determined in this study was used to determine cell density in the cultures (Supplementary Figure 3). Growth rate (μ) was calculated between the individual incubation periods (*t*_1_ and *t*_2_) with an assumption of exponential growth; i.e., μ (h^-1^) = (ln *N*_t2_ – ln *N*_t1_)/(*t*_2_ -*t*_1_), where *N*_t1_ and *N*_t2_ are the cell densities. On the basis of the growth rate, *T*_min_, *T*_opt_, and *T*_max_ of the strains were determined.

## Results and Discussion

### 16S rRNA Gene Sequences and Growth Temperature Estimation

A total of 16 clones were randomly selected from a clone library of *H. hispanica* strain JCM8911, and the sequences of 16S rRNA genes were determined (1440 bp). Three types of 16S rRNA genes were identified and matched *rrsA*, *rrsB*, and *rrsC* in the genome sequence as determined by Liu et al. (2011). The sequences of *rrsB* and *rrsC* were 99.6% identical, whereas the sequences of *rrsA* and *rrsBC* were 94.6–94.9% identical. These results confirm previous reports of intragenomic polymorphism of 16S rRNA genes in *Haloarcula* (e.g., Cui et al., 2009).

The sequence regions between the archaea-specific primers 109aF and 915aR (795 bp) were selected from the 16S rRNA gene sequences. The *P*_GC_ of the internal sequences of *rrsA*, *rrsB*, and *rrsC* were 58.9, 56.5, and 56.4%, respectively (**Table 2**). The offset between the *P*_GC_ of *rrsA* and *rrsBC* was ∼2.5%. **Table 2** summarizes the estimated growth temperatures based on these *P*_GC_ values. The estimated *T*_min,_ *T*_opt_, and *T*_max_ based on the *P*_GC_ of *rrsA* were 32.6 ± 16.7, 51.6 ± 11.8, and 59.7 ± 13.1°C, which are much higher than those calculated from the *P*_GC_ of *rrsB* and *rrsC*. The offsets between the estimated growth temperatures based on *P*_GC_ of *rrsA* and *rrsBC* was >10°C. These findings may indicate that harboring 16S rRNA genes with relatively high- and low-*P*_GC_ values allows *H. hispanica* to maintain rapid growth over a wide temperature range.

**Table 2:** Actual growth temperatures, 16S rRNA genes, and estimated growth temperatures of *Haloarcula hispanica* JCM8911.

Actual growth temperature^a^			16S rRNA gene			Estimated growth temperature^b^		
*T*_min_ (°C)	*T*_opt_ (°C)	*T*_max_ (°C)	Accession no.	Type	*P*_GC_ (%)	*T*_min_ (°C)	*T*_opt_ (°C)	*T*_max_ (°C)
25	45	50	LC085245	*rrsA*	58.9	32.6 ± 16.7	51.6 ± 11.8	59.7 ± 13.1
			LC085246	*rrsB*	56.5	22.2 ± 16.4	39.8 ± 11.6	48.1 ± 12.8
			LC085247	*rrsC*	56.4	21.7 ± 16.4	39.3 ± 11.5	47.6 ± 12.8

^a^*Actual growth temperatures were determined in this study*.

^b^*Estimated growth temperatures were calculated from *P*_GC_ of *rrsA*, *rrsB*, and *rrsC* using the microbial molecular thermometer proposed by Kimura et al. (2013)*.

### Survey of Expression of 16S rRNA Genes of *H. hispanica*

To check the selectivity of the specific primers designed in this study, qPCR was performed with PCR products of *rrsA*, *rrsB*, and *rrsC* that were amplified from the clones in the 16S rRNA gene-clone library. The *rrsA*-specific primer set, rrsAf/rrsAr, provided the proper products from only diluted PCR products of *rrsA* as templates at annealing temperature of 58°C, whereas the *rrsBC*-specific primer set, rrsBCf/rrsBCr, provided the proper products from only diluted PCR products of *rrsB* and *rrsC* at annealing temperature of 58°C. These results indicated that these primer sets were sufficiently selective to detect and quantify *rrsA* and *rrsBC*, respectively.

Our survey of 16S RNA gene expression demonstrated that *rrsA* and *rrsBC* expression varied with temperatures. In particular, the expression ratio of *rrsA* to *rrsBC* (*rrsA*:*rrsBC*) increased with culture temperature (**Figure 1**). The ratios at 45 and 50°C exceeded 1.0, which were significantly greater than those in the 25–35°C range (*P* < 0.05 by Student’s *t*-test). On the other hand, the ratios at 25, 30, and 35°C were below 1.0. Especially, the ratios at 25 and 30°C were 0.56, which means that total expression of *rrsB* and *rrsC* was almost as twice as that of *rrsA* at the low temperatures. Our results suggest that transcription of high-*P*_GC_ 16S rRNA gene *rrsA* and low-*P*_GC_ 16S rRNA genes *rrsBC* may be regulated in response to culture temperature.

### Construction and Culture of rRNA Operon Double-Mutants

We constructed rRNA operon single-mutant strains (HA2, HB2, and HC2) that lack *rrnA*, *rrnB*, or *rrnC*, respectively (Supplementary Table 1). We further constructed rRNA operon double-mutant strains, namely HCB2 harboring only *rrnA* and HAB2 harboring only *rrnC* (Supplementary Table 1). Operon deletion was confirmed by PCR with specific primer sets and by electrophoresis of the PCR products (Supplementary Figure 4).

The wild-type *H. hispanica* was able to grow at temperature ranging from 25 to 50°C, with optimum growth at 45°C (**Table 2**). The fastest growth was 0.16 h^-1^ at the *T*_opt_ of 45°C (Supplementary Figure 5). The growth characteristics of the wild-type strain at various temperatures were almost the same as those of the rRNA operon single-mutant strains (data not shown). In the culture experiments using the double-mutant strains, the wild-type grew faster than HCB2 and HAB2 at all tested temperatures (**Figure 2**). HCB2 and HAB2 were able to grow within the same temperature range (i.e., 25–50°C) as the wild-type strain (Supplementary Figure 5). The HCB2 that harbors only *rrnA* containing high-*P*_GC_ *rrsA* grew optimally at 45°C. On the other hand, optimum growth of HAB2, which harbors only *rrnC* containing low-*P*_GC_ *rrsC*, was slightly shifted to low temperature of 40°C.

At 25°C, the wild-type *H. hispanica* grew significantly faster than HAB2 and HCB2 (**Figure 2A**). Condon et al. (1995) used *Escherichia coli* with multiple rRNA operons inactivated by antibiotic cassettes to demonstrate that the number of rRNA operons in the genome affects cell proliferation rate. Yano et al. (2013) found that the existence of multiple rRNA operons underlies the high growth rate of *Bacillus subtilis*. Another study reported that the copy number of rRNA operons on the genomes is correlated with growth rate of the prokaryotes under optimal culture condition (Vieira-Silva and Rocha, 2010; Roller et al., 2016). Our results support these studies and suggest that the number of rRNA operons influences cell proliferation rate at low temperatures close to actual *T*_min_ (25°C).

At 30°C, HCB2 had slightly higher growth rate than HAB2 (**Figure 2B**), and this difference increased in cultures at 35 and 40°C (**Figures 2C,D**). At 45 and 50°C, the growth curves for HCB2 were almost identical with those of the wild-type strain (**Figures 2E,F**). These findings suggest that the rRNAs transcribed from *rrnA*, including the high-*P*_GC_ 16S rRNA gene *rrsA*, result in more rapid growth of *H. hispanica* at high temperatures of 45 and 50°C. The expression survey in this study also showed that expression of *rrsA* was higher than those of *rrsBC* at 45 and 50°C (**Figure 1**), which also supports that rRNAs transcribed from *rrnA* including high-*P*_GC_ 16S rRNA gene *rrsA* may be important for growth under the high temperature conditions.

### Survival Strategy of *Haloarcula* Strains

*Haloarcula hispanica* has been identified in a number of solar salterns and desert salt lakes (e.g., Arahal et al., 1996; Pašić et al., 2005; Tapilatu et al., 2010; Mani et al., 2012). These hyper-saline habitats generally have large daily temperature fluctuations, i.e., temperature can vary by >10°C (Post, 1977; Wieland et al., 2005; Sima et al., 2013; Andrade et al., 2015). Because of this habitat feature, it is predicted that *H*. *hispanica* would express the rRNA operon with high-*P*_GC_ 16S rRNA gene to grow faster in the daytime, when environmental temperatures rise to around *T*_max_ (50°C). On the other hand, *H. hispanica* would express all of the three rRNA operons to grow in the nighttime and/or early morning, when environmental temperatures drop to around *T*_min_ (25°C).

Fifteen *Haloarcula* strains have been isolated from hyper-saline environments worldwide (e.g., Juez et al., 1986; Ihara et al., 1997; Oren et al., 1999; Yang et al., 2007). Except for *H. aidinensis*, 14 *Haloarcula* strains so far examined harbor several different 16S rRNA gene sequences in the genome (Supplementary Table 3). Thus, intragenomic 16S rRNA gene heterogeneity seems to be common feature in the genus *Haloarcula*. We further confirmed that 9 of the 14 *Haloarcula* strains for which sequence was available show a >2.0% difference in *P*_GC_ among the 16S rRNA genes. The estimated growth temperatures based on *P*_GC_ values of the respective 16S rRNA genes suggested >10°C differences as well as *H. hispanica* (Supplementary Table 3). Additionally, previous study using a *Haloarcula* strain suggested that the sequences of putative promoter regions were obviously different among upstream regions of rRNA operons (Dennis et al., 1998; Dennis, 1999; López-López et al., 2007). These findings suggest that *Haloarcula* strains may regulate the expression of these 16S rRNA genes in response to culture temperature conditions, and this can be tested in future studies.

## Conclusion

In this study, we determined the sequences and *P*_GC_ values of 16S rRNA genes in the genome of the halophilic archaeon *H*. *hispanica*, and growth temperatures of *H*. *hispanica* were estimated based on the *P*_GC_ values. The estimated growth temperatures of cells carrying the high-*P*_GC_ 16S rRNA gene (*rrsA*) were approximately 10°C higher than those carrying the low-*P*_GC_ 16S rRNA genes (*rrsB* and *rrsC*), suggesting that *H. hispanica* harbors different 16S rRNA genes of different *P*_GC_ to maintain rapid growth in a wide range of temperatures.

We characterized the expression of *rrsA* and *rrsBC* of *H. hispanica* at different growth temperatures. We found that *rrsA* was expressed at significantly higher levels than *rrsBC* at higher temperatures such as 45 and 50°C. Our results indicate the importance of a high-*P*_GC_ 16S rRNA gene at raised growth temperatures in the *Haloarcula* species. We further constructed rRNA operon double-mutant strains of *H. hispanica*. Culture experiments showed that the wild-type strain grew faster than the mutant strains at temperatures between 25 and 40°C. At 45 and 50°C, the double-mutant strain harboring only *rrnA* (including *rrsA*) grew much faster than the double-mutant strain harboring only *rrnC* (including *rrsC*), and the growth rate was similar to that of the wild-type strain. These findings suggest that the copy number of rRNA operons affects the growth rate of *H. hispanica* under low temperature conditions and that rRNAs transcribed from *rrnA*, which contains the high-*P*_GC_ 16S RNA gene *rrsA*, function to promote rapid growth under high temperature conditions.

## Author Contributions

YS and HK conceived this study. YS performed all the experiments and drafted the manuscript. TF helped YS to construct the mutant strains. All authors confirmed and approved the final manuscript.

## Conflict of Interest Statement

The authors declare that the research was conducted in the absence of any commercial or financial relationships that could be construed as a potential conflict of interest.
