# Supplementary material for: Expression and Function of Different Guanine-Plus-Cytosine Content 16S rRNA Genes in Haloarcula hispanica at Different Temperatures
Source: Front Microbiol. 2017 Mar 28;8:482. doi: 10.3389/fmicb.2017.00482 (PMC5368182; doi:10.3389/fmicb.2017.00482)
Supplement: Supplementary file 1 [file Data_Sheet_1.pdf]

frontiers

Supplementary Material

Expression and function of different guanine-plus-cytosine content 16S rRNA genes in *Haloarcula hispanica* at different temperatures

Yu Sato[1], Taketomo Fujiwara[1,2], Hiroyuki Kimura[1,3,4*]

[1] Department of Environment and Energy Systems, Graduate School of Science and Technology, Shizuoka University, Shizuoka, Japan

[2] Department of Biological Science, Faculty of Science, Shizuoka University, Shizuoka, Japan

[3] Department of Geosciences, Faculty of Science, Shizuoka University, Shizuoka, Japan

[4] Research Institute of Green Science and Technology, Shizuoka University, Shizuoka, Japan

***Correspondence:**

Hiroyuki Kimura

Department of Geosciences, Faculty of Science, Shizuoka University, Shizuoka, Japan

836 Oya, Suruga-ku, Shizuoka 422-8529, Japan

E-mail address: kimura.hiroyuki@shizuoka.ac.jp

Tel: +81-54-238-4784

Fax: +81-54-238-0491

Supplementary Material and Methods

Disruption of rRNA operons in *Har. hispanica*

Standard protocols for handling *E. coli* and *Hfx. volcanii* DNA were followed, with slight modifications, and the double-integration method was adapted to construct *rrn*-deleted mutants (Dyall-Smith, 2009; Sambrook and Russell, 2001; Tu et al., 2005). First, specific PCR primer sets were designed using Genetyx Mac ver. 17.0.6 (Genetyx) and NCBI's Primer Blast to amplify each operon, including the regions ~1000 bp upstream and downstream (Supplementary Figures 1 and 2). The primer sets were named AUF/ADR for the region containing *rrnA*, BUF/BDR for the region containing *rrnB*, and CUF/CDR for the region containing *rrnC* (Supplementary Table 2). Additionally, primer sets were designed to amplify only the upstream and downstream regions of each rRNA operon with the intervening vector sequence (Supplementary Figures 1 and 2). These primer sets were named ADF/AUR, BDF/BUR, and CDF/CUR.

The genomic regions containing rRNA operons were amplified by PCR using the primer sets AUF/ADR, BUF/BDR, and CUF/CDR. The PCR products were ligated into vector pCR4Blunt-TOPO (Life Technologies). The ligated plasmids were transformed into *E. coli* TOP10 cells (Life Technologies), and clone libraries were constructed as described above. Next, the regions other than the rRNA operon in the plasmid, i.e., the downstream and upstream regions and vector, were amplified using the primer sets ADF/AUR, BDF/BUR, and CDF/CUR. A novobiocin-resistance gene derived from pMDS2 was ligated with the PCR products, generating plasmids pNA, pNB, and pNC (Holmes and Dyall-Smith, 1991; Supplementary Table 1). Additionally, a mevinolin (a statin)-resistance gene derived from pWL102 was ligated with the PCR products, generating pMA, pMB, and pMC (Lam and Doolittle, 1989; Supplementary Table 1). The resistance gene–ligated plasmids were transformed into *E. coli* TOP10 cells (Life Technologies), and clone libraries were constructed as described above. The plasmids were extracted from individual clones and then purified for transformation.

Transformation and isolation of mutant strains were performed on Medium 168, which contained 2 g casamino acid, 2 g Bacto yeast extract, 1 g sodium glutamate, 3 g trisodium citrate, 20 g $MgSO_4 \cdot 7H_2O$, 2 g KCl, 200 g NaCl, 36 mg $FeCl_2 \cdot 4H_2O$, 0.36 mg $MnCl_2 \cdot 4H_2O$, and 20 g agar per liter of distilled water. Plasmid pNA, pNB, or pNC was introduced into the wild-type *Har. hispanica* by the PEG600 method (Cline and Doolittle, 1992; Dyall-Smith, 2009). Pop-in strains, indicated by colonies on Medium 168 supplemented with 10 $\mu g\ ml^{-1}$ of novobiocin (Wako, Osaka,

Japan), were collected. The obtained colonies were designated as HA1 (for pNA), HB1 (for pNB), and HC1 (for pNC) (Supplementary Table 1 and Supplementary Figure 1). HA1, HB1, and HC1 were streaked on Medium 168 without novobiocin for a second homologous recombination event. Pop-out colonies appearing on Medium 168 were collected. Actual disruption of *rrnA*, *rrnB*, and *rrnC* was confirmed by PCR amplification using primer sets AVF/ACR, BVF/BCR, and CVF/CCR, which are complementary to primer sets AUR/ADF, BUR/BDF, and CUR/CDF, respectively (Supplementary Table 2). The mutant strains, which lacked *rrnA*, *rrnB*, and *rrnC*, were designated as HA2, HB2, and HC2, respectively (Supplementary Table 2).

We further constructed rRNA operon double-mutant strains harboring only one of the three operons, *rrnA*, *rrnB*, or *rrnC*. For disruption of the second rRNA operon, pMB, pMA (or pMC), and pMB was introduced into HC2, HC2 (or HA2), and HA2 with PEG600 as described above (Supplementary Figure 2). These strains were grown on Medium 168 supplemented with 100 μg ml^{-1} of simvastatin (another statin; Wako). The pop-in strains with colonies appearing on the medium were designated HCB1, HCA1 (or HAC1), and HAB1 (Supplementary Table 1). Each strain was separately streaked on Medium 168 without simvastatin for the second homologous recombination. The colonies appeared on the medium were collected, and disruption of operons *rrnA*, *rrnB*, and *rrnC* was confirmed by PCR amplification using primer sets AVF/ACR, BVF/BCR, and CVF/CCR, respectively (Supplementary Table 2). The rRNA operon double-mutant strains harboring only *rrnA*, *rrnB*, or *rrnC* were designated as HCB2, HCA2 (or HAC2), and HAB2, respectively (Supplementary Table 1).

Supplementary Table 1. Plasmids and *Haloarcula hispanica* strains used in this study.

Plasmid or strain	Description or genotype		Source or reference
**Plasmid**			
pCR4Blunt-TOPO	Plasmid with ampicillin-resistance gene and kanamycin-resistance gene		Invitorgen
pMDS2	Plasmid with novobiocin-resistance gene (Nov^R)		Holmes and Dyall-Smith (1991)
pWL102	Plasmid with mevinolin-resistance gene (Mev^R)		Lam and Doolittle (1989)
pNA	Plasmid with *rrnA* adjacent fragment of *Har. hispanica* and nov^R for disruption of *rrnA*		This study
pNB	Plasmid with *rrnB* adjacent fragment of *Har. hispanica* and nov^R for disruption of *rrnB*		This study
pNC	Plasmid with *rrnC* adjacent fragment of *Har. hispanica* and nov^R for disruption of *rrnC*		This study
pMA	Plasmid with *rrnA* adjacent fragment of *Har. hispanica* and mev^R for disruption of *rrnA*		This study
pMB	Plasmid with *rrnB* adjacent fragment of *Har. hispanica* and mev^R for disruption of *rrnB*		This study
pMC	Plasmid with *rrnC* adjacent fragment of *Har. hispanica* and mev^R for disruption of *rrnC*		This study
**Strain**			
JCM8911	Wild-type strain	JCM8911	JCM
HA1	*rrnA*+::[Δ*rrnA*-nov^R]	JCM8911, pNA pop-in	This study
HA2	Δ*rrnA* including nov^R	JCM8911, pNA pop-out	This study
HB1	*rrnB*+::[Δ*rrnB*-nov^R]	JCM8911, pNB pop-in	This study
HB2	Δ*rrnB* including nov^R	JCM8911, pNB pop-out	This study
HC1	*rrnC*+::[Δ*rrnC*-nov^R]	JCM8911, pNC pop-in	This study
HC2	Δ*rrnC* including nov^R	JCM8911, pNC pop-out	This study
HCB1	Δ*rrnC rrnB*+::[Δ*rrnB*-mev^R]	HC2, pMB pop-in	This study
HCB2	Δ*rrnC*Δ*rrnB* including nov^R and mev^R	HC2, pMB pop-out	This study
HCA1	Δ*rrnC rrnA*+::[Δ*rrnA*-mev^R]	HC2, pMA pop-in	This study
HCA2	Δ*rrnC*Δ*rrnA* including nov^R and mev^R	HC2, pMA pop-out	Not obtained
HAC1	Δ*rrnA rrnC*+::[Δ*rrnC*-mev^R]	HA2, pMC pop-in	This study
HAC2	Δ*rrnA*Δ*rrnC* including nov^R and mev^R	HA2, pMC pop-out	Not obtained
HAB1	Δ*rrnA rrnB*+::[Δ*rrnB*-mev^R]	HA2, pMB pop-in	This study
HAB2	Δ*rrnA*Δ*rrnB* including nov^R and mev^R	HA2, pMB pop-out	This study

JCM, Japan Collection of Microorganisms

Supplementary Table 2. Designed primers used for construction of mutant strains in this study

Name	Description	Position on two chromosomes	Target
AUF	5'-GAA CCG GTA CGT GAT GGC TTC AA-3'	I; 212084-212106	upstream region of *rrnA*
ADR	5'-ACC GTC CGG AGA TAC ACA GGT TGA-3'	I; 219338-219315	downstream region of *rrnA*
BUF	5'-CAC CCA CCT GTT CGA GTA TCT-3'	I; 1774700-1774720	upstream region of *rrnB*
BDR	5'-GTA CGC TGA TCT CGG TGT CA-3'	I; 1782143-1782124	downstream region of *rrnB*
CUF	5'-TAT CAG AGC CAG ACC TGA GT-3'	II; 7556-7575	upstream region of *rrnC*
CDR	5'-CTG TCG GCA GGT GAA ATA GT-3'	II; 14991-14972	downstream region of *rrnC*
ADF	5'-GAA CGA ATT CAC AGC ACT CCT CGA-3'	I; 218315-218338	downstream region of *rrnA*
AUR	5'-GTT AGG CGG GAT CAC ACC CAT ATA-3'	I; 213140-213117	upstream region of *rrnA*
BDF	5'-ATT CCA TCT TGA CGG CGG AC-3'	I; 1781031-1781050	downstream region of *rrnB*
BUR	5'-ATT CCA GTC TAT GCG GCA GA-3'	I; 1775752-1775733	upstream region of *rrnB*
CDF	5'-ACT GAC CCA CTC AGT GAA CA-3'	II; 13928-13947	downstream region of *rrnC*
CUR	5'-TAC ACG AAC ACA AAG CCT CA-3'	II; 8686-8667	upstream region of *rrnC*
AVF	5'-TAT ATG GGT GTG ATC CCG CCT AAC-3'	I; 213117-213140	*rrnA* or drug resistance gene
ACR	5'-TCG AGG AGT GCT GTG AAT TCG TTC-3'	I; 218338-218315	*rrnA* or drug resistance gene
BVF	5'-TCT GCC GCA TAG ACT GGA AT-3'	I; 1775733-1775752	*rrnB* or drug resistance gene
BCR	5'-GTC CGC CGT CAA GAT GGA AT-3'	I; 1781050-1781031	*rrnB* or drug resistance gene
CVF	5'-TGA GGC TTT GTG TTC GTG TA-3'	II; 8667-8686	*rrnC* or drug resistance gene
CCR	5'-TGT TCA CTG AGT GGG TCA GT-3'	II; 13947-13928	*rrnC* or drug resistance gene

Supplementary Table 3. P_{GC} **of 16S rRNA genes (*rrs*) and estimated growth temperatures for *Haloarcula* strains.**

	16S rRNA gene					Estimated growth temperature[d]			
Strain[a]	Type	Accession number	Sequnece difference[b] (%)	P_{GC} (%)	P_{GC} offset[c] (%)	T_{min} (ºC)	T_{opt} (ºC)	T_{max} (ºC)	T_{opt} offset[e] (ºC)
Haloarcula hispanica JCM8911	*rrsA*	LC085245	5.4	58.9	2.5	32.6 ± 16.7	51.6 ± 11.8	59.7 ± 13.1	12.3
	rrsB	LC085246		56.5		22.2 ± 16.4	39.8 ± 11.6	48.1 ± 12.8	
	rrsC	LC085247		56.4		21.7 ± 16.4	39.3 ± 11.5	47.6 ± 12.8	
Haloarcula marismrotui	*rrsA*	AY596297	5.6	58.6	2.4	31.7 ± 16.7	50.2 ± 11.8	58.4 ± 13.0	11.9
	rrsB	AY596298		56.2		20.9 ± 16.4	38.3 ± 11.5	46.6 ± 12.8	
	rrsC	AY596297		56.5		22.2 ± 16.4	39.8 ± 11.6	48.1 ± 12.8	
"*Haloarcula* sp. CBA1115"	*rrsA*	CP010529	5.4	59.0	2.9	33.1 ± 16.8	52.2 ± 11.8	60.3 ± 13.1	14.4
	rrsB	CP010529		56.1		20.4 ± 16.4	37.8 ± 11.5	46.1 ± 12.8	
	rrsC	CP010529		56.1		20.4 ± 16.4	37.8 ± 11.5	46.1 ± 12.8	
"*Haloarcula hispanica* N601"	*rrsA*	CP006884	5.2	58.9	2.4	32.6 ± 16.7	51.6 ± 11.8	59.7 ± 13.1	11.9
	rrsB	CP006884		56.5		22.2 ± 16.4	39.8 ± 11.6	48.1 ± 12.8	
	rrsC	CP006885		56.5		22.2 ± 16.4	39.8 ± 11.6	48.1 ± 12.8	
Haloarcula amylolytica	*rrsA*	DQ826512	5.6	58.5	2.4	30.9 ± 16.7	49.7 ± 11.8	57.9 ± 13.0	11.9
	rrsB	DQ826513		56.1		20.4 ± 16.4	37.8 ± 11.5	46.1 ± 12.8	
	rrsC	DQ826518		56.5		22.2 ± 16.4	39.8 ± 11.6	48.1 ± 12.8	
Haloarcula japonica	*rrsA*	EF645684	5.0	58.4	2.2	30.5 ± 16.7	49.2 ± 11.7	57.4 ± 13.0	10.9
	rrsB	EF645685		56.2		20.9 ± 16.4	38.3 ± 11.5	46.6 ± 12.8	
	rrsC	EF645686		56.4		21.7 ± 16.4	39.3 ± 11.5	47.6 ± 12.8	
"*Haloarcula rubripromontorii* SL3"	*rrsA*	KU198854	4.4	58.9	2.2	32.6 ± 16.7	51.6 ± 11.8	59.7 ± 13.1	10.8
	rrsB	KU198855		56.7		23.0 ± 16.4	40.8 ± 11.6	49.0 ± 12.8	
Haloarcula quadrata	*rrsA*	AB010965	5.0	58.6	2.1	31.7 ± 16.7	50.2 ± 11.8	58.4 ± 13.0	10.4
	rrsB	AB010964		56.5		22.2 ± 16.4	39.8 ± 11.6	48.1 ± 12.8	
Haloarcula argentinensis	*rrsA*	EF645680	4.8	58.5	2.0	30.9 ± 16.7	49.7 ± 11.8	57.9 ± 13.0	9.9
	rrsB	EF645681		56.5		22.2 ± 16.4	39.8 ± 11.6	48.1 ± 12.8	
"*Haloarcula californiae*"	*rrsA*	AB477985	5.4	58.4	1.7	30.5 ± 16.7	49.2 ± 11.7	57.4 ± 13.0	8.4
	rrsB	AB477984		56.7		23.0 ± 16.4	40.8 ± 11.6	49.0 ± 12.8	
Haloarcula vallismortis	*rrsA*	EF645687	5.3	58.4	1.3	30.5 ± 16.7	49.2 ± 11.7	57.4 ± 13.0	6.4
	rrsB	EF645688		57.1		24.8 ± 16.5	42.8 ± 11.6	51.0 ± 12.9	
"*Haloarcula sinaiiensis*"	*rrsA*	D14130	2.7	57.4	1.0	26.1 ± 16.5	44.3 ± 11.6	52. 5± 12.9	5.0
	rrsB	D14129		56.4		21.7 ± 16.4	39.3 ± 11.5	47.6 ± 12.8	
Haloarcula tradensis	*rrsA*	FJ429313	3.9	57.8	0.7	27.9 ± 16.6	46.2 ± 11.7	54.4 ± 13.0	3.4
	rrsB	FJ429314		57.1		24.8 ± 16.5	42.8 ± 11.6	51.0 ± 12.9	
	rrsC	FJ429316		57.4		26.1 ± 16.5	44.3 ± 11.6	52.5 ± 12.9	
Haloarcula salaria	*rrsA*	FJ429318	1.7	56.8	0.3	23.5 ± 16.5	41.3 ± 11.6	49.6 ± 12.8	1.5
	rrsB	FJ429317		56.5		22.2 ± 16.4	39.8 ± 11.6	48.1 ± 12.8	
"*Haloarcula aidinensis*"	*rrsA*	AB771438	0	58.6	0	31.7 ± 16.7	50.2 ± 11.8	58.4 ± 13.0	0
	rrsB	AB771439		58.6		31.7 ± 16.7	50.2 ± 11.8	58.4 ± 13.0	

[a]Quotation marks represent six strains that their names have not been published validly.

[b]Sequence difference between *rrsA* and *rrsB* or *rrsC*.

[c]Offsets between P_{GC} of *rrsA* and P_{GC} of *rrsB* or *rrsC*.

[d]Growth temperatures estimated from P_{GC} of *rrsA* , *rrsB*, and *rrsC* based on microbial molecular thermometer of Kimura et al. (2013).

[e]Offsets between optimum growth temperatures estimated from P_{GC} of *rrsA* and that estimated from P_{GC} of *rrsB* or *rrsC*, respectively.

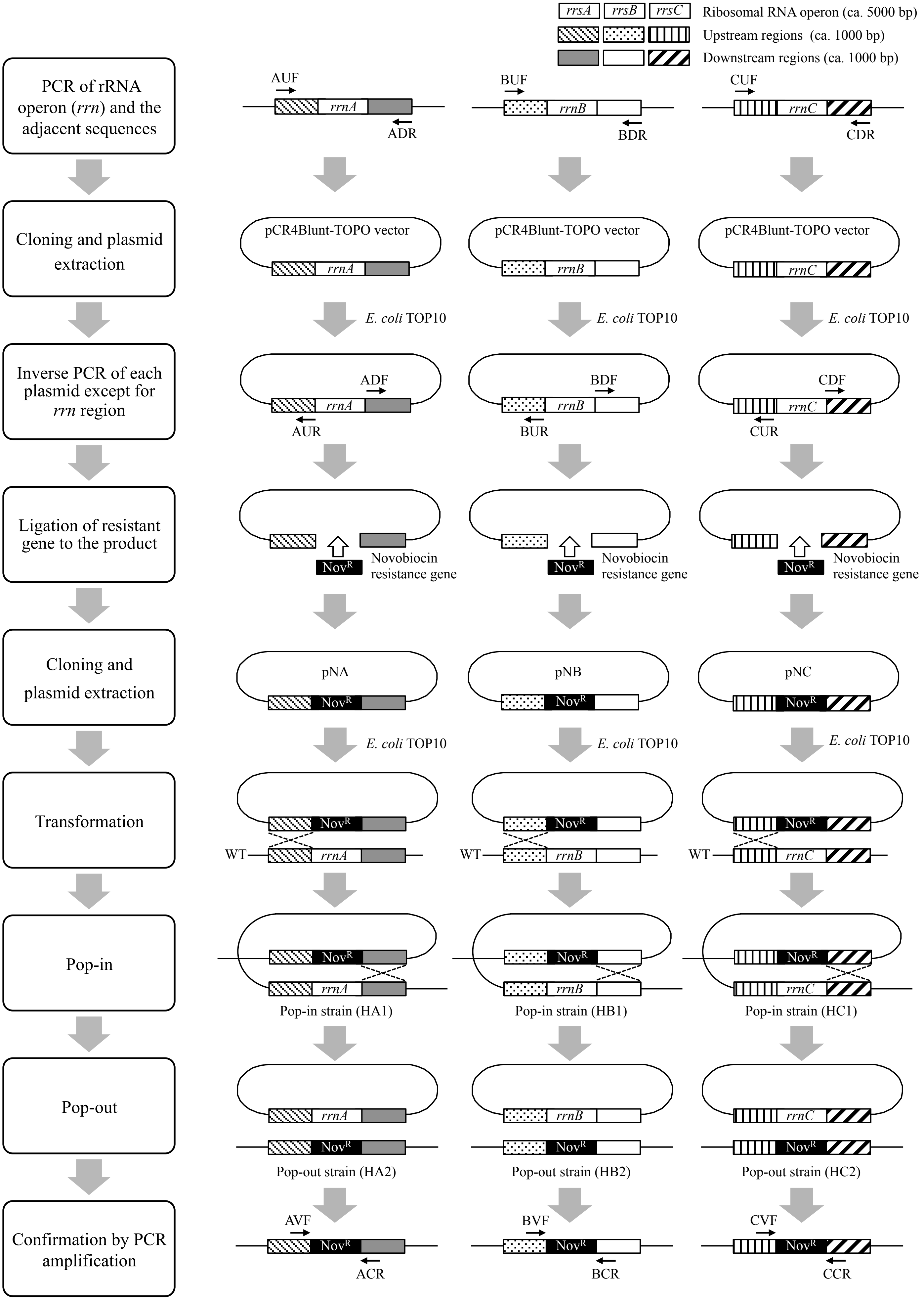

Supplementary Figure 1. Flow chart of the first rRNA operon (*rrn*) deletion method. Mutant strains, plasmids, and primer sets designed in this study are summarized in Supplementary Tables 1 and 2.

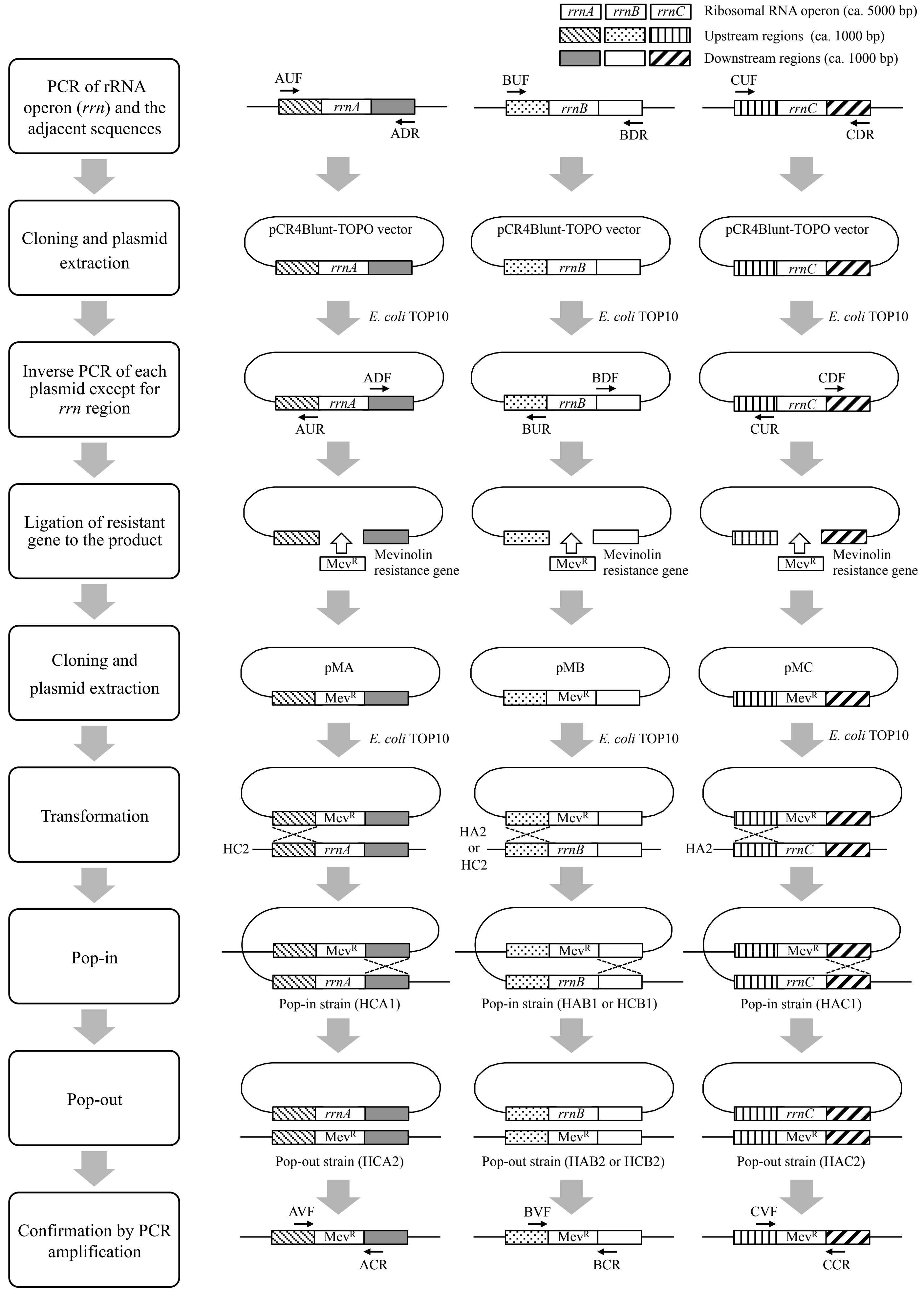

Supplementary Figure 2. Flow chart of the second rRNA operon (*rrn*) deletion method. Mutant strains, plasmids, and primer sets designed in this study are summarized in Supplementary Tables 1 and 2.

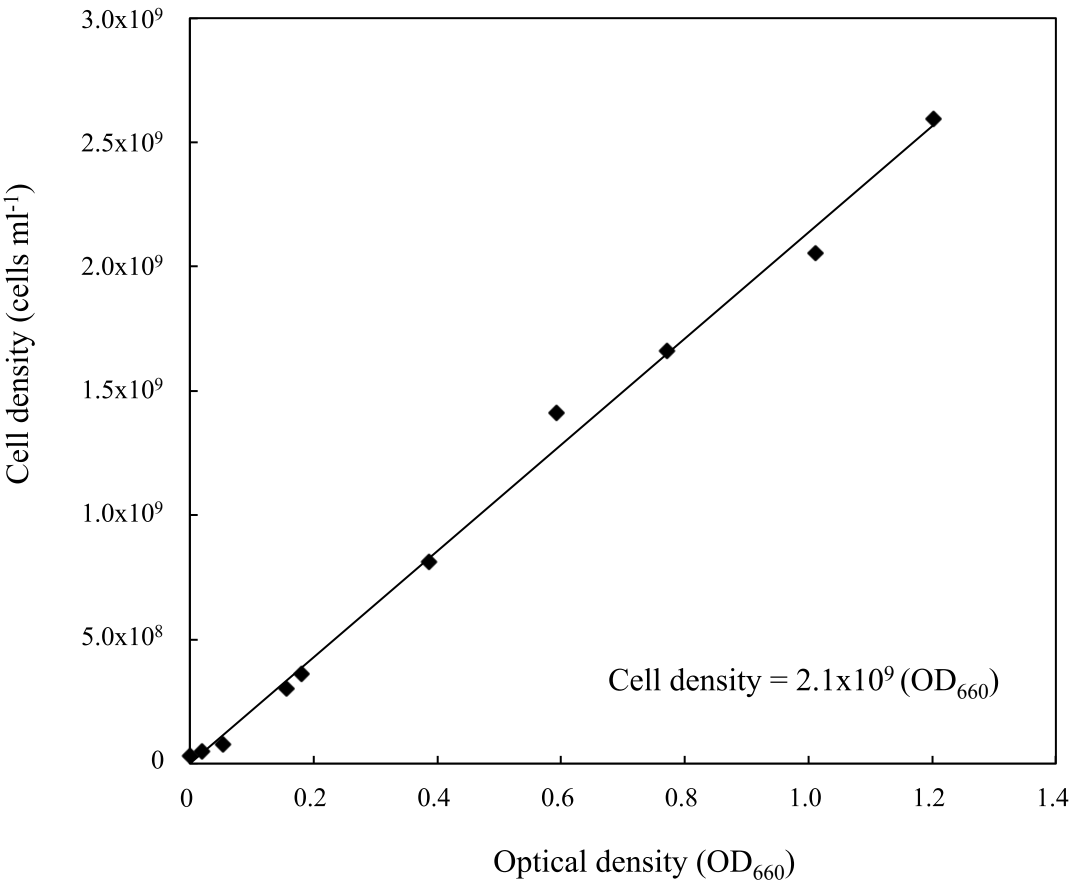

Supplementary Figure 3. Correlation between cell density and OD_{660} value in *Haloarcula hispanica*.

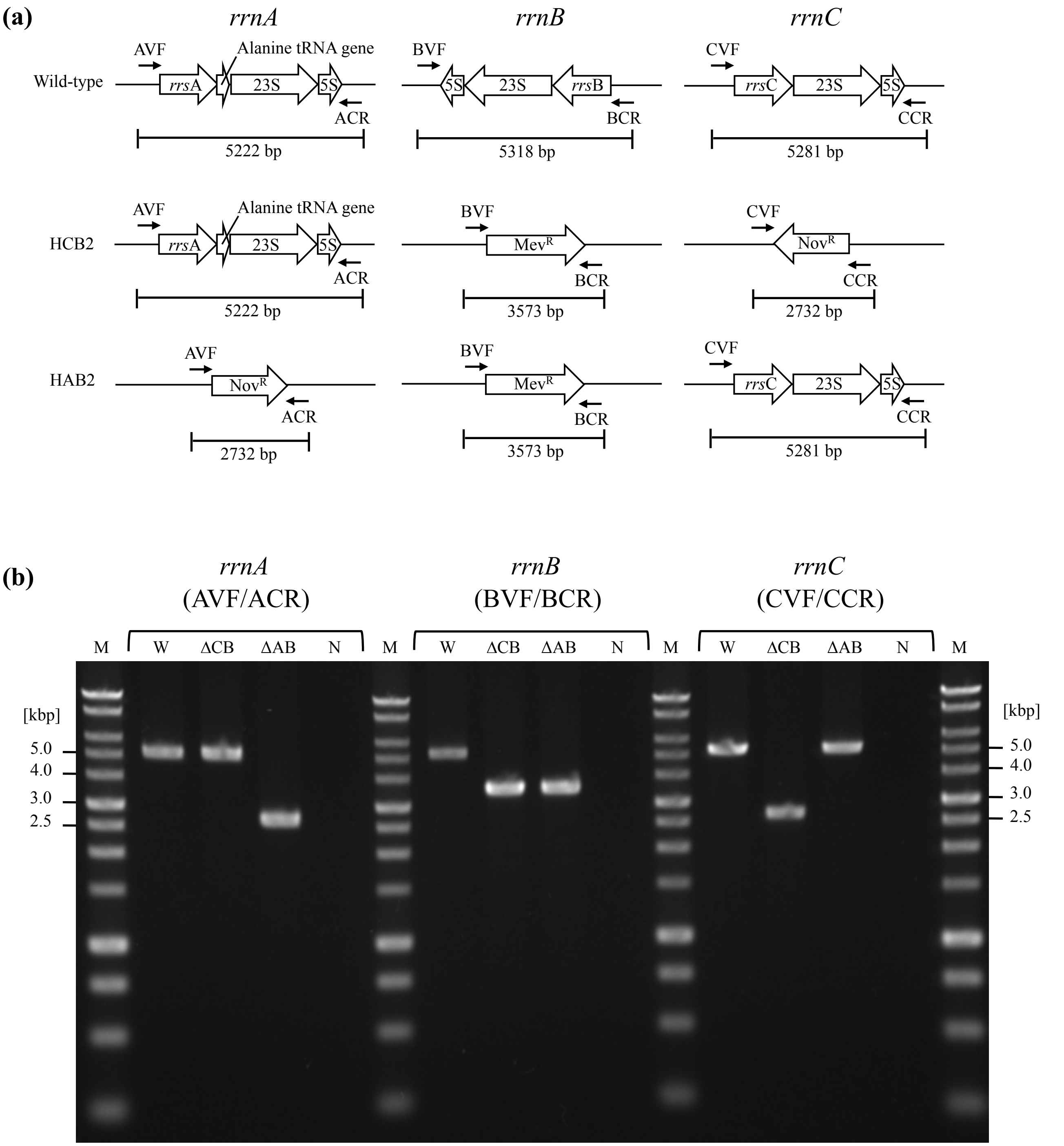

Supplementary Figure 4. Confirmation of rRNA operon deletion in mutant strains of *Har. hispanica*. (a) Location of PCR amplification and the length of each PCR product (scale bars). (b) Electrophoresis results for the PCR products of deletion sites from both wild-type (W) and mutant strains HCB2 (ΔCB) and HAB2 (ΔAB). N: negative control. M: markers, 1-kb DNA ladder (Promega, Madison, WI, USA).

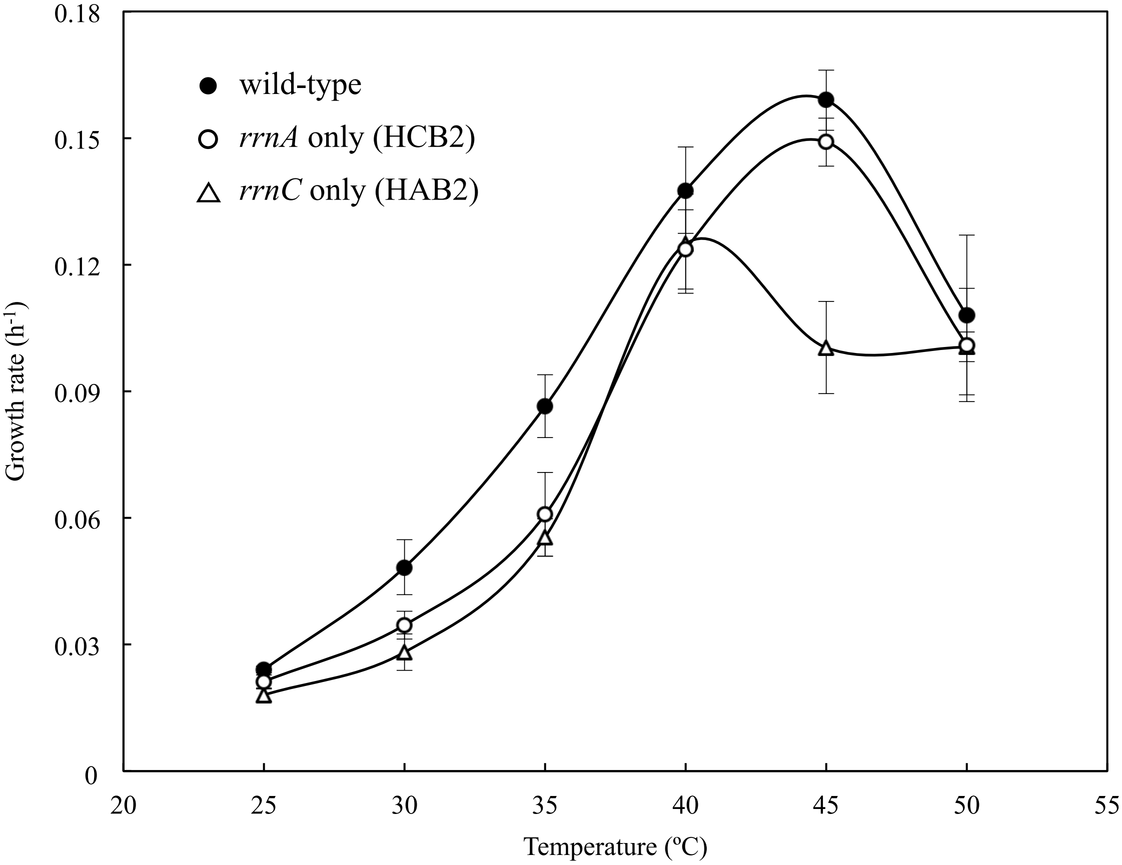

Supplementary Figure 5. Maximum growth rates (μ) of wild-type, HCB2, and HAB2 at each temperature. Error bars denote standard deviation of mean values for quadruplicate or quintplicate measurements.

Supplementary References

Dyall-Smith, M. L. (2009). *The Halohandbook - protocols for haloarchaeal genetics* ver. 7.2. Available online at: http://www.haloarchaea.com/resources/halohandbook

Holmes, M. L., and Dyall-Smith, M. L. (1991). Mutations in DNA gyrase result in novobiocin resistance in halophilic archaebacteria. *J. Bacteriol.* 173, 642–648.

Kimura, H., Mori, K., Yamanaka, T., and Ishibashi, J. (2013). Growth temperatures of archaeal communities can be estimated from the guanine-plus-cytosine contents of 16S rRNA gene fragments. *Environ. Microbiol. Rep.* 5, 468–474. doi: 10.1111/1758-2229.12035

Lam, W. L., and Doolittle, W. F. (1989). Shuttle vectors for the archaebacterium *Halobacterium volcanii*. *Proc. Natl. Acad. Sci. USA* 86, 5478-5482. doi: 10.1073/pnas.86.14.5478

Sambrook, J., and Russell, D. W. (2001). *Molecular Cloning.* Cold Spring Harbor, NY: Cold Spring Harbor Laboratory.

Tu, D., Blaha, G., Moore, P. B., and Steitz, T. A. (2005). Gene replacement in *Haloarcula marismortui*: construction of a strain with two of its three chromosomal rRNA operons deleted. *Extremophiles* 9, 427–435. doi: 10.1007/s00792-005-0459-y
